# Sacroiliites infectieuses dans le centre tunisien: étude rétrospective de 25 cas

**DOI:** 10.11604/pamj.2016.24.3.8659

**Published:** 2016-05-03

**Authors:** Foued Bellazreg, Zeineb Alaya, Zouhour Hattab, Nadia Ben Lasfar, Mohamed Laziz Ben Ayeche, Elyes Bouajina, Amel Letaief, Wissem Hachfi

**Affiliations:** 1 Service de Maladies Infectieuses, CHU Farhat Hached, Sousse, Tunisie; 2 Service de Rhumatologie, CHU Farhat Hached, Sousse, Tunisie; 3 Service d'Orthopédie, CHU Sahloul, Sousse, Tunisie

**Keywords:** Sacroiliites infectieuses, bactéries pyogènes, brucellose, tuberculose, Infectious sacroiliitis, pyogenic bacteria, brucellosis, tubercolosis

## Abstract

Les sacroiliites infectieuses sont rares mais peuvent se compliquer de séquelles fonctionnelles invalidantes. Décrire les caractéristiques cliniques et bactériologiques des sacroiliites infectieuses chez les patients suivis à Sousse, Centre Tunisien. Etude rétrospective, descriptive, des cas de sacroiliites infectieuses chez les patients hospitalisés à Sousse entre 2000 et 2015. Le diagnostic a été retenu devant des signes cliniques, d'imagerie, et microbiologiques évocateurs. Vingt-cinq patients, 10 hommes et 15 femmes, d’âge moyen 41 ans (19-78) ont été inclus. Les sacroiliites étaient dues à des bactéries pyogènes dans 14 cas (56%), brucelliennes dans 6 cas (24%), et tuberculeuses dans 5 cas (20%). La durée moyenne d’évolution était de 61, 45 et 402 jours respectivement. Les signes cliniques les plus fréquents étaient les douleurs fessières (92%) et la fièvre (88%). La radiographie standard était anormale dans 75% des cas. La TDM et l'IRM sacro-iliaques dans tous les cas. Le diagnostic a été confirmé bactériologiquement dans 24 cas (96%). La durée moyenne d'antibiothérapie était de 83 jours dans les sacroiliites à pyogènes, et de 102 jours dans les SI brucelliennes. L’évolution était favorable chez 12 patients (48%), 9 patients (36%) ont gardé une douleur sacro-iliaque séquellaire, et 4 patients (16%) sont décédés. Dans notre étude, la durée d’évolution de la sacroiliite infectieuse ne permettait pas de prédire la bactérie responsable, d'où la nécessité d'obtenir une documentation bactériologique afin de prescrire une antibiothérapie appropriée.

## Abstract (en)

Infectious sacroiliitis are rare but they can be complicated by disabling functional sequelae. To describe the clinical and bacteriological characteristics of infectious sacroiliitis among patients treated in Sousse Medical Center, Tunisia. A retrospective, descriptive study, of infectious sacroiliitis among patients hositalized in Sousse between 2000 and 2015. The diagnosis was made on the basis of medical signs, imaging, microbiological indicators. In the study were enrolled twenty five patients, 10 men and 15 women; the average age was 41 years (19-78). Sacroiliitis were due to pyogenic bacteria in 14 cases (56%), brucella bacteria in 6 cases (24%) and tuberculosis bacteria in 5 cases (20%). The mean duration of symptoms was 61, 45 and 402 days respectively. The most common clinical signs were buttock pain (92%) and fever (88%). Standard radiographic evaluation was abnormal in 75% of cases. CT scan and MRI of the sacroiliac joints was performed in all cases. The diagnosis was bacteriologically confirmed in 24 cases (96%). The average duration of antibiotic treatment was 83 days in the pyogenic sacroiliitis, and 102 days in brucellar sacroiliitis. The evolution was favorable in 12 patients (48%), 9 patients (36%) had sequelae of sacroiliac joint pain and 4 patients (16%) died. In our study, time frame of infectious sacroiliitis evolution did not predict the causative bacterium, hence the need for bacteriological documentation in order to prescribe appropriate antibiotic therapy.

## Introduction

Les articulations sacro-iliaques peuvent être le siège de plusieurs pathologies avec risque de séquelles fonctionnelles parfois invalidantes. A côté des spondylarthropathies qui représentent l’étiologie habituelle des sacro-iliites bilatérales, une étiologie infectieuse doit être recherchée devant toute sacro-iliite unilatérale [1]. Les sacroiliites infectieuses (SII) sont rares. Elles représentent 1 à 2% des arthrites septiques à pyogènes, 26 à 60% des localisations ostéo-articulaires de la brucellose, et 3 à 9,7% des tuberculoses ostéo-articulaires [2–6]. L'objectif de cette étude était de décrire les aspects cliniques, microbiologiques et thérapeutiques des SII prises en charge à Sousse, Centre Tunisien.

## Méthodes

Il s'agit d'une étude rétrospective, descriptive, des cas de SII chez les patients âgés de plus de 15 ans, hospitalisés dans les services de Maladies Infectieuses, de Rhumatologie, et d'Orthopédie de Sousse entre janvier 2000 et juillet 2015. Le diagnostic de SI a été retenu devant des signes cliniques, biologiques et d'imagerie évocateurs. Le diagnostic de SI à pyogènes a été retenu en cas d'isolement d'une bactérie dans l'articulation sacro-iliaque, le pus d'abcès, ou un liquide biologique normalement stérile (sang, urines…). Le diagnostic de SI brucellienne a été retenu devant une sérologie de Wright positive à un titre = 1/160. Le diagnostic de SI tuberculeuse a été retenu devant la présence de bacilles acido-alcoolo-résistants ou l'isolement de *Mycobacterium tuberculosis* dans des prélèvements de l'articulation sacro-iliaque, d'un abcès profond, ou d'une autre localisation tuberculeuse, ou devant des signes anatomopathologiques évocateurs. Les données ont été saisies et analysées sur le logiciel SPSS, version 10. Les variables qualitatives ont été exprimées en pourcentages et les variables quantitatives en moyennes.

## Résultats

### Données cliniques

**Figure 1:**
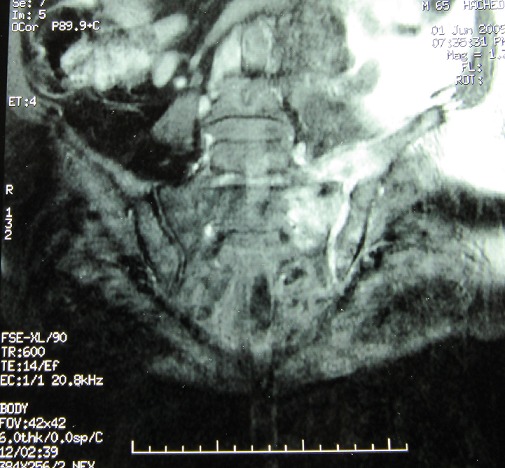
IRM des sacro-iliaques (séquence STIR): élargissement de l'interligne sacro-iliaque gauche avec érosions corticales et irrégularité des surfaces articulaires

Vingt-cinq patients ont été hospitalisés pour SII, dont 16 (64%) en Maladies Infectieuses, 7 (28%) en Rhumatologie et 2 (8%) en Orthopédie. Il s'agissait de 10 hommes et 15 femmes (sex-ratio 0,66), d’âge moyen 41 ans (19-78). Les SII étaient dues à des bactéries pyogènes dans 14 cas (56%), brucelliennes dans 6 cas (24%) et tuberculeuses dans 5 cas (20%). Tous les cas étaient confirmés bactériologiquement hormis 2 cas de SI tuberculeuse. Un contact avec les ovins, bovins, ou caprins était noté chez 5 patients (85%) ayant une SI brucellienne, et un comptage tuberculeux était noté chez 2 patients (40%) ayant une SI tuberculeuse. Le délai diagnostic moyen était de 124 jours (6-730). Cette durée était de 61 jours (6-300) dans les SI à pyogènes, de 45 jours (15-120) dans les SI brucelliennes et de 402 jours (300-730) dans les SI tuberculeuses. Vingt-trois patients (92%) avaient des douleurs fessières qui étaient unilatérales dans 18 cas (78%) et de type inflammatoire dans 22 cas (96%). Les autres signes cliniques fréquents étaient la fièvre (22 cas, 88%), l'impotence fonctionnelle (20 cas, 80%), et les rachialgies lombaires (9 cas, 36%). Vingt-trois patients (92%) avaient un syndrome inflammatoire biologique. Une hyperleucocytose supérieure à 15000/mm3 était notée seulement dans 3 cas (21%) de SI à pyogènes. La radiographie des sacro-iliaques, réalisée chez 16 patients (68%), était anormale dans 12 cas (75%). La TDM et l'IRM sacro-iliaques, réalisées chez 48% et 28% des patients respectivement étaient anormales dans tous les cas. Les anomalies les plus fréquentes étaient l’érosion osseuse (92%) (Figure 1), un abcès des parties molles (86%) et un épanchement intra-articulaire (71%). Une ou plusieurs autres localisations infectieuses étaient associées à la sacroiliite chez 15 patients (60%). Il s'agissait essentiellement d'abcès du psoas dans 6 cas (40%), de spondylodiscite dans 4 cas (27%) et d'arthrite de la symphyse pubienne dans 3 cas (20%).

### Données bactériologiques

Les SII étaient dues à des bactéries pyogènes dans 14 cas (56%), brucelliennes dans 6 cas (24%) et tuberculeuses dans 5 cas (20%). Les SI à pyogènes étaient dues à Staphylococcus aureus dans 8 cas (57%), Escherichia coli dans 3 cas (22%), *Klebsiella pneumoniae, Klebsiella oxytoca* et *Pseudomonas aeruginosa* dans un cas (7%) chacun. Le diagnostic était posé par les examens microbiologiques suivants: hémocultures (7 cas, 50%), examen bactériologique d'un prélèvement de l'articulation sacro-iliaque dans (4 cas, 29%), ECBU (2 cas, 15%), examen bactériologique d'un abcès du muscle fessier (2 cas, 15%), et prélèvement cutané (1 cas, 7%). Une porte d'entrée était retrouvée chez 8 patients (58%). Il s'agissait d'une lésion cutanée dans 3 cas (Staphylococcus aureus), vasculaire dans 2 cas (Staphylocoque aureus et *Klebsiella pneumoniae)*, urinaire dans 2 cas (Escherichia coli) et digestive dans 1 cas *(Klebsiella oxytoca)*. Le diagnostic des SI brucelliennes était posé par la positivité de la sérologie de Wright dans tous les cas. Les hémocultures, réalisées chez un seul patient, étaient négatives. Le diagnostic des SI tuberculeuses était confirmé microbiologiquement dans 4 cas (examen direct et culture positifs dans 2 cas, examen direct négatif et culture positive dans 2 cas). L'intradermoréaction (IDR) à la tuberculine, réalisée chez 4 patients, était positive dans 2 cas (50%).

### Traitement

Dans les SI à pyogènes (n = 14), l'antibiothérapie initiale était administrée par voie intraveineuse (IV) dans tous les cas, pendant une durée moyenne de 19 jours (14-21). Un relais oral était prescrit dans 12 cas (deux patients sont décédés à J10 et à J21 d’évolution), pendant une durée moyenne de 64 jours (30-77). La durée moyenne totale d'antibiothérapie était de 83 jours (60-98). Les antibiotiques les plus fréquemment prescrits étaient l'oxacilline (7 cas, 50%) et la céfotaxime (3 cas, 22%) par voie IV; la ciprofloxacine (7 cas, 50%), l'acide fusidique (4 cas, 29%) et le cotrimoxazole (4 cas, 29%) par voie orale. Un drainage percutané écho-guidé des abcès des parties molles était réalisé chez un patient, et un traitement chirurgical de la SI était indiqué chez une patiente de 66 ans porteuse d'une SI à Staphylocoque aureus avec volumineux abcès pelviens non améliorés après 3 semaines d'antibiothérapie appropriée. Dans les SI brucelliennes, tous les patients étaient traités par l'association doxycyline-rifampicine pendant une durée moyenne de 102 jours (60-150). Dans les SI tuberculeuses, un patient ayant une tuberculose disséminée est décédé à J10 d'hospitalisation d'un syndrome de défaillance multiviscérale, et 4 patients étaient traités par l'association isoniazide-rifampicine- pyrazinamide- ethambutol pendant 2 mois avec relais par l'association isoniazide-rifampicine. Parmi ces 4 patients, une patiente est décédée d'une tuberculose neuro-méningée à 4 mois de traitement, une patiente, traitée pendant une durée totale de 18 mois, a bien évolué, et deux patientes sont encore sous isoniazide-rifampicine avec nette amélioration clinique. Une corticothérapie était prescrite chez 3 patients ayant une tuberculose neuro-méningée dans 2 cas et disséminée dans 1 cas. Quatre patients (/25, 16%) sont décédés. Les causes de décès étaient un hématome rétropéritonéal suite à un traitement chirurgical d'une SI à Staphylococcus aureus, un choc septique à Escherichia coli chez un patient ayant un myélome multiple, une tuberculose disséminée, et une tuberculose neuroméningée. Les autres patients (n = 21) étaient suivis pendant une période moyenne de 22 mois (3-168). Une guérison sans séquelles était obtenue chez 12 patients (48%) et 9 patients (36%%) ont gardé une douleur sacro-iliaque séquellaire. Aucune récidive n'a été notée.

## Discussion

Dans notre étude, les SII ont été observées le plus souvent chez des adultes jeunes avec prédominance féminine et le délai diagnostic était très variable. Dans des études tunisiennes, françaises et sud-africaines, l’âge moyen des patients variait entre 27 et 39,7 ans, les sex-ratios entre 0,15 et 0,69, et les signes cliniques les plus fréquents étaient la fièvre, la boiterie et la douleur sacro-iliaque, souvent unilatérale. Le délai diagnostic variait de quelques semaines dans les SI à pyogènes et les SI brucelliennes à plusieurs mois dans les SI tuberculeuses [1, 7–11]. La durée d’évolution a seulement une valeur d'orientation puisqu'une évolution de plusieurs mois peut aussi être observée dans les SI à pyogènes. La radiographie standard peut être normale à un stade précoce. Les signes radiologiques apparaissent avec un délai de l'ordre de 15 jours pour les SI à pyogènes, et de 15 à 30 jours pour les SI brucelliennes et tuberculeuses [12]. La radiographie standard était anormale dans 60,6% à 100% des cas dans deux études tunisiennes et une étude française [7, 8, 10], et dans 75% des cas dans notre étude. La TDM et surtout l'IRM sont plus sensibles que la radiographie standard et montrent des anomalies de l'articulation sacro-iliaque et des parties molles adjacentes [13, 14]. Dans notre étude, ces deux examens ont été réalisés dans 48% et 28% des cas respectivement et avaient montré des anomalies dans tous les cas. Ainsi, le premier examen à réaliser devant une suspicion de SII est l'IRM, et à défaut la TDM. La plupart des études publiées sur les SII concernent des cas isolés ou un faible nombre de cas ou ne contiennent pas des données bactériologiques détaillées [2, 9, 13]. Les deux plus grandes études ont été réalisées en France sur 39 cas de SI à pyogènes et en Tunisie sur 22 cas de SI tuberculeuses [8, 10]. Dans toutes ces études, la quasi-totalité des cas étaient dues à des bactéries pyogènes, à Brucella et à *Mycobacterium tuberculosis*. De rares cas de SI fongiques, à Candida ou à*Cryptococcus neoformans* ont été rapportés [7, 15, 16]. Dans notre étude, tous les cas de SII étaient dus à des bactéries pyogènes, à une brucellose ou à une tuberculose. Les SI à pyogènes étaient confirmées microbiologiquement dans tous les cas et les bactéries les plus fréquemment isolées étaient Staphylococcus aureus et les entérobactéries. Dans une étude Française, une confirmation microbiologique a été obtenue dans 77% des cas, et les bactéries les plus fréquemment isolées étaient Staphylococcus aureus (53%), Staphylococcus à coagulase négative (17%), les streptocoques (17%) et Pseudomonas aeruginosa (10%) [13]. Les moyens diagnostiques les plus fréquents étaient le prélèvement de l'articulation SI (53%) et les hémocultures (47%). Ainsi, les bactéries les plus fréquemment responsables des SI à pyogènes sont les staphylocoques, les entérobactéries et les streptocoques. D'autres bactéries étaient plus rarement responsables de SII. Il s'agit de pneumocoque, Salmonella, *Haemophilus influenzae* et Clostridium difficile [15, 17–23].

Dans la brucellose, les tests sérologiques sont faciles à réaliser et très sensibles (98,9% à 100%), alors que les hémocultures sont moins sensibles (11,2 à 19,8%) [4, 24]. Dans notre étude, tous les cas ont été confirmés par la sérologie de Wright. Dans les SI tuberculeuses, la confirmation bactériologique est obtenue dans 45% à 53% des cas, et un aspect anatomopathologique évocateur a été noté dans 32 à 88% des cas. L'IDR à la tuberculine était positive dans 86% des cas [8, 9]. Dans notre étude, une confirmation bactériologique a été obtenue dans 80% des cas, et l'IDR à la tuberculine était positive dans 50% des cas. La difficulté d'avoir une confirmation bactériologique de la tuberculose sacro-iliaque s'explique par le caractère paucibacillaire de la tuberculose ostéo-articulaire et de la tuberculose extra- pulmonaire en général, d'où l'intérêt de réunir des arguments épidémiologiques, cliniques et para-cliniques pour le diagnostic de SI tuberculeuse. Il n'existe pas de recommandations pour l'antibiothérapie des SII. Par analogie avec les recommandations françaises et anglaises pour le traitement des spondylodiscites infectieuses à pyogènes, il semble raisonnable de proposer une antibiothérapie initiale par voie IV pendant 2 à 3 semaines, suivie d'une antibiothérapie par voie orale, avec une durée totale de 6 à 12 semaines [25, 26]. En l'absence de documentation bactériologique, il est recommandé de prescrire une antibiothérapie anti-staphylococcique et, en cas d’échec, une antibiothérapie active sur les entérobactéries [25]. Dans la brucellose, les deux schémas thérapeutiques de référence sont l'association doxycycline pendant 6 semaines associée à la streptomycine pendant 2 à 3 semaines, et l'association doxycycline-rifampicine pendant 6 semaines [27]. La durée du traitement recommandée dans la brucellose ostéo-articulaire était de 6 à 12 semaines dans une étude et d'au moins 3 mois dans une autre étude [4, 23]. Dans la tuberculose ostéo-articulaire, l'Organisation Mondiale de la Santé recommande une durée totale de 9 mois, dont 2 mois de quadrithérapie initiale par isoniazide-rifampicine-pyrazinamide-éthambutol relayée par isoniazide-rifampicine [28]. Dans une étude Tunisienne, la durée totale moyenne du traitement des SI tuberculeuses était de 9 mois [8]. Dans notre étude, la durée moyenne de l'antibiothérapie des SI à pyogènes était de 81 jours, dont 19 jours d'antibiothérapie initiale par voie IV, et les SI brucelliennes étaient traitées par l'association doxycycline-rifampicine pendant une durée moyenne de 15 semaines (8-21). L'antibiothérapie seule est souvent suffisante dans le traitement des SII. Cependant, le recours à la chirurgie est nécessaire en cas d’échec de l'antibiothérapie. Cet échec est fréquent en cas de volumineux abcès ou d'ostéomyélite avec séquestres et nécrose. Le traitement chirurgical consiste en un débridement des tissus infectés, un drainage des volumineux abcès, ou, en cas de présence de gros séquestres, en une arthrodèse [2, 29, 30]. Dans une étude Tunisienne, une intervention chirurgicale a été réalisée chez 45% des patients [8]. Dans notre étude, une intervention chirurgicale a été indiquée dans un seul cas (4%). L'intervention s'est compliquée d'un hématome rétropéritonéal avec état de choc hémorragique et décès de la patiente. Dans une étude française sur les SI à pyogènes, 2,5% des patients sont décédés et 54% ont gardé des douleurs résiduelles [10]. Dans une étude tunisienne sur les SI tuberculeuses, ces taux étaient de 0% et 20% respectivement [8]. Dans notre étude, ces taux étaient de 16% et 36% respectivement. Ainsi le pronostic des SII semble essentiellement fonctionnel, marquée par la persistance possible de douleurs résiduelles. Le décès est souvent en rapport avec une infection systémique.

## Conclusion

Dans notre étude, la durée d’évolution de la sacroiliite infectieuse ne permettait pas de prédire la bactérie responsable, d'où la nécessité d'obtenir une documentation bactériologique afin de prescrire une antibiothérapie appropriée.

### Etat des connaissances actuelle sur le sujet

- Les manifestations cliniques et à l'imagerie des sacroiliites infectieuses (SII);
- Les microorganismes responsables des SII

### Contribution de notre étude à la connaissance

- Les microorganismes responsables de SII dans le centre Tunisien;
- Un nombre relativement important de patients (plus d'une vingtaine). A notre connaissance, la plupart des articles publiés sur les SII consistent en des case reports ou en des séries de moins de 10 cas.
